# Binary and Ternary Nanocomposite Membranes for Gas Separation Incorporating Finely Dispersed Carbon Nanotubes in a Polyether Block Amide Matrix

**DOI:** 10.3390/polym17030314

**Published:** 2025-01-24

**Authors:** Danilo Vuono, Gabriele Clarizia, Daniela Clotilde Zampino, Paola Bernardo

**Affiliations:** 1 Institute on Membrane Technology (ITM-CNR), 87036 Rende, Italy; 2 Institute of Polymers, Composites and Biomaterials (IPCB-CNR), 95126 Catania, Italy

**Keywords:** multiwalled carbon nanotubes, Pebax^®^2533, membrane, gas separation

## Abstract

This work addressed the fine dispersion of Multiwalled Carbon Nanotubes (MWCNTs) in a polymer matrix to obtain Mixed Matrix Membranes (MMMs) suited for gas separation. Not-purified MWCNTs were effectively loaded within a polyether block amide (Pebax^®^2533) matrix, up to 24 wt%, using ultrasonication as well as a third component (polysorbate) in the dope solution. The obtained flexible thin films were investigated in terms of morphology, thermal properties, characterized by SEM, FT-IR, DSC, TGA, and gas permeation tests. The response to temperature variations of gas permeation through these nanocomposite specimens was also investigated in the temperature range of 25–55 °C. Defect-free samples were successfully obtained even at a significantly high loading of CNTs (up to 18 wt%), without a pre-treatment of the fillers. A remarkable enhancement of gas permeability upon the nanocarbons loading was reached, with a threshold value at a loading of ca. 7 wt%. The addition of polysorbates in the ternary MMMs further improves the dispersion of the filler, enhancing also the permselectivity of the membrane.

## 1. Introduction

Carbon nanotubes (CNTs) have unique properties, such as remarkable electrical conductivity, good thermal conductivity, significant mechanical tensile strength, and light weight. They were observed by Radushkevich et al. [1] in the middle 1950s and by Oberlin et al. [2] as by-products of synthesis from aromatic compounds, but the first systematic studies on these materials date back to 1991 with Ijima [3]. Currently, CNTs are available in bulk quantities, more than a thousand tons per year [4]. The manufacture capacity of Multiwalled Carbon Nanotubes (MWCNTs) exceeds that of Single Wall Carbon Nanotubes (SWNTs), due to a less sophisticated synthesis of MWNTs, compared to SWNTs, that results in a reduced cost [5]. The MWNT production is experiencing a renewed growth, driven by the increasing demand for conductive materials to be used in lithium-ion batteries for energy storage applications [6]. Moreover, MWCNTs are also investigated for the cathodes in sodium-ion batteries to improve the electronic conductivity [7].

CNTs are widely used as fillers in polymeric composites to produce lightweight materials [8]. Representative examples include the production of reinforced substitutes of metals in automotive parts. Nanocomposites based on a polymer matrix loaded with CNTs were studied as membranes for gas separation [9,10].

Polyether block amides (Pebax^®^) are plasticizer-free Thermoplastic Elastomers (TPEs) [11] that were successfully used as polymer matrices to prepare membranes for gas separation, demonstrating a preferential permeation for polar molecules such as CO_2_ [12]. Being rubbery, these polymers were exploited to simply prepare nanocomposite membranes (Mixed Matrix Membranes, MMMs) incorporating different types of additives (e.g., solid nanoparticles, Ionic Liquids, etc.) [13,14,15]. Different studies explored the addition of CNTs to the Pebax^®^1657 matrix [16,17,18], evidencing improved gas permeation properties with respect to the pristine Pebax matrix. Compared to silica and polystyrene colloid fillers, SWNTs or MWNTs (in the range 0–7 wt%) enhanced the gas permeability of Pebax^®^1657 and maintained the polymer selectivity, reaching a maximum in the separation performance at a loading of 5 wt% [17]. A progressive increase in permeability is reported for MWCNTs dispersed in the same polymer in a larger loading range (up to 35 wt%) [19].

**Figure 1:**
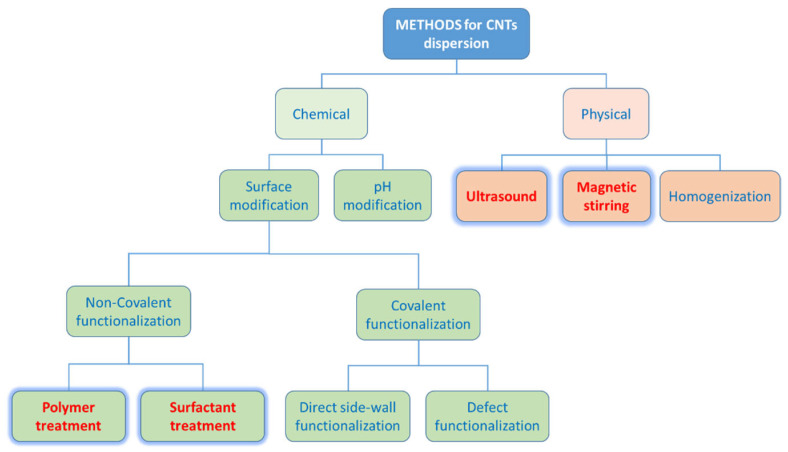
Methods for the CNTs dispersion (adapted from [24]). Those adopted in this work are highlighted in red.

A crucial aspect in the development of such nanocomposite membranes is the filler dispersion in the matrix that depends on the processing methodology. In this respect, the solution mixing performs better than the melt compounding [20]. The combination of mechanical stirring and ultrasonication can lead to a relatively uniform dispersion of nanofillers within a polymeric solution. After the solvent evaporation, polymer macromolecules cover the fillers that will be included within the matrix [21]. However, CNTs tend to form aggregates, owing to their hydrophobic character that hampers a good dispersion in solvents as well as in viscous polymer melts. Polymers [22] and surfactants [23] are effectively used in the non-covalent surface modification approach to exfoliate the CNT bundles and disperse them (Figure 1).

Ternary systems that comprise solid nanoparticles, a polymer matrix and a coupling agent represent a successful approach to improve the compatibility between different phases. MMMs based on Pebax and comprising CNTs as fillers and glycerol triacetate (GTA) as a third component were developed for CO_2_ separation [19]. Another study, describing the preparation of MMMs containing functionalized MWCNTs mixed with a non-ionic surfactant (Triton X100) in Pebax^®^1657, reported a performance above the Robeson’s upper bound for CO_2_/N_2_ separation of the membranes containing 4 wt% of MWCNTs [25]. Triton X100 showed the highest dispersing power for CNTs compared to Tween 20, Tween 80, and sodium dodecyl sulfate (SDS) by virtue of its benzene ring [26], due to the pi-stacking interaction [27]. However, since 2021, octylphenol ethoxylates as Triton X100 are included in the REACH list of contaminants of emerging concern by the European Chemicals Agency (ECHA) [28] due to the action on wildlife of its degradation products (hormone-like activity) [29].

In the present study, nanocomposite membranes suited for gas separation were prepared by doping a matrix of Pebax^®^2533 with MWCNTs that were loaded up to a concentration of 24 wt% in order to improve the gas separation performance of the pristine polymer. The Pebax^®^2533 grade has been less explored as matrix to prepare MMMs for the gas separation, due to its lower permselectivity compared to that of the Pebax^®^1657 grade. However, its larger amount of the flexible polyether block results in enhanced permeation fluxes and makes it more suited to host nanofillers. In order to avoid time-consuming and costly procedures, not purified MWCNTs were used.

In previous works [30], we demonstrated that the addition of polysorbates, which are non-ionic surfactants with a low toxicity profile, to solutions of Pebax^®^1657 resulted in membranes with enhanced gas permeability. Interesting permeability enhancement was also proved for Pebax^®^2533/polysorbate membranes [31]. Therefore, to favor the polymer/nanocarbon compatibilization, two polysorbates (i.e., sorbitan monooleate T80 and sorbitan monolaurate T20) were also employed in the membrane fabrication, producing ternary MMMs. T80, used in the preparation of MWNTs nanofluids, resulted in a good stability over time compared to other surfactants [32].

The main transport parameters through all these types of nanocomposite membranes as well as morphological and thermal properties were evaluated.

## 2. Materials and Methods

**Figure 2:**
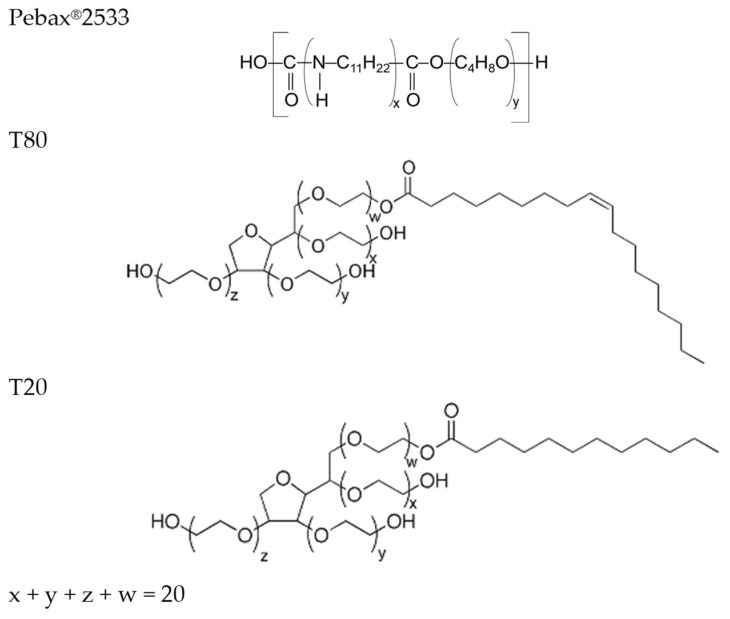
Chemical structure of the polymer and the polysorbate (T20, T80) surfactants.

The block copolymer Pebax^®^2533, composed by 80% of poly (tetramethylene oxide) and 20% of polyamide 12, was received from Arkema, Milan (Italy). Tween 20 (T20) and Tween 80 (T80) Polysorbates (Sigma Aldrich, Milan, Italy) were used as dispersants for membrane preparation (Figure 2). Non-purified Multiwalled Carbon Nanotubes (MWCNT, from Nanocyl S. A., Sambreville, Belgium) were used as fillers. Ethanol (absolute, VWR, Milan, Italy) was used as a solvent for preparing the polymer solution.

Gasses used for permeation tests (He, O_2_, N_2_, CH_4_ and CO_2_), having a purity of 99.99+%, were purchased from SAPIO, Monza, Italy.

### 2.1. Membrane Preparation

Nanocomposite films were prepared according to a procedure of solution blending. Dense isotropic films based on the neat polymer were prepared and used as a reference. The polymer pellets were dissolved into ethanol under reflux conditions at a concentration of 3 wt%. Weighted amounts of the fillers were sonicated in ethanol for 1 h, mixed to the polymer solution, and then the ultrasonication was repeated. An ultrasonication bath operating at 45 kHz (USC 300 TH, VWR) was used. The treatment on the CNT suspension was prolonged for 1 h and 30 min in the case of the CNT suspension mixed to the polymeric solution.

Fixed amounts of the dope solution were cast within stainless-steel rings using a glass plate as support for MMMs and a Teflon plate for neat samples. After the controlled evaporation of the solvent, the films were detached from the support and cut for the characterization. The final samples are denoted as *C-x* where *x* represents the nanofiller amount in wt% (i.e., 0, 3, 6, 9, 12, 18, 21 and 24 wt%).

Another series of membranes was prepared according to the above described procedure by adding different amounts of T80 or T20 to the sonicated MWCNTs dispersion in ethanol. The obtained slurry was furtherly sonicated, then it was mixed to the polymeric solution and sonicated again.

The CNT concentration in the prepared membranes (expressed in wt%) was calculated using the respective weight amount of the membrane components on a dry basis, according to Equation (1a) for the binary samples and Equation (1b) for the ternary membranes:

(1a) $$\mathrm{binary}\;x_{CNT}=\frac{w_{CNT}}{w_{CNT}+w_{Polymer}}\cdot 100$$

(1b) $$\mathrm{ternary}\;x_{CNT}=\frac{w_{CNT}}{w_{CNT}+w_{Polymer}+w_{Surfactant}}\cdot 100$$

The sample codes for the ternary MMMs are those reported in Table 1. The ternary samples were indicated using the prefix C-12 since all these samples were prepared with a CNT/(polymer + CNT) ratio of 12 wt% (see Equation (1a)). However, by adding increasing amounts of the third component (i.e., the surfactant), the CNT percentage referred to the total dry weight (polymer + surfactant + CNT) is lower than 12%, ranging from 4.5 to 8 wt% (see Equation (1b)).

### 2.2. Fourier Transform Infrared Spectroscopy (FT-IR)

FT-IR analysis was carried out on the membranes in the Attenuated Total Reflection (ATR) mode using a Spectrum One (Perkin Elmer, Milan, Italy). The spectra were recorded with a resolution of 4 cm^−1^ (16 scans) in the region 650–4000 cm^−1^. The neat CNTs were analyzed using the same instrument in the transmission mode at room conditions by preparing KBr pellets with the CNT powder in a hydraulic press.

### 2.3. Thermal Analysis

Thermogravimetric analysis (TGA) was carried out on the prepared films using a Q500 apparatus (TA Instruments, Milan, Italy) under a nitrogen atmosphere. The samples were dried and loaded into a platinum pan (sample weight from 4 mg to 6 mg). TGA data and their derivatives (DTG) were recorded by increasing the temperature from 40 to 600 °C at a heating rate of 10 °C/min.

Calorimetric measurements were carried out on neat polymer and CNT-loaded films using a differential scanning calorimeter (DSC, TA Instruments Q100), comprising a liquid sub ambient accessory. Before measurements, the instrument was calibrated using high-purity standards (indium and cyclohexane). The analyses were carried out using nitrogen as purge gas at a rate of 10 °C/min. The samples were initially equilibrated at −90 °C. A first heating scan from −90 °C to 200 °C was carried out to erase the previous thermal history of the samples. It was followed by a cooling run to −90 °C and by a second heating one from −90 °C to 200 °C.

The crystallinity degree in the two copolymer blocks was calculated from the enthalpy of fusion of the corresponding crystalline domains:

(2) $$X_{c,i}=\frac{{∆H}_{m,i}}{w_{i}\;\cdot \;{∆H}_{m,i}^{0}}\cdot 100$$

where Δ*H*_m_ stands for the fusion heat of the microphase, *w*_i_ represents the weight fraction of the considered phase (polyamide or polyether, respectively), and ${∆H}_{m}^{0}$ is the fusion heat of the 100% crystalline microphase (246 J g^−1^ for PA12 and196.6 J g^−1^ for PTMO, respectively [33]).

### 2.4. Gas Permeation Tests

The gas permeation properties were analyzed in a constant-volume/variable-pressure device, using circular samples of the prepared membranes (effective area in the range 2.14–11.3 cm^2^). The membrane thickness was determined as the average of multiple measurements taken in different points of the sample using a digital micrometer (Metric Dial Indicator 543-561D, Mitutoyo, Lainate (MI), Italy).

The experimental apparatus, described elsewhere [34], comprises a turbomolecular pump as a last stage to accurately evacuate the membrane sample before each test, removing traces of residual solvent, humidity, and previously tested gasses. The increasing gas pressure signal in the permeate side was monitored vs. time, elaborating the data according to the time-lag method [35]. This procedure enables the determination of the permeability (*P*) and the diffusion coefficient (*D*) of each gas through the membrane. The gas solubility (*S*) was indirectly evaluated as *S* = *P*/*D*, according to the “solution-diffusion” model [36].

The ideal selectivity was calculated by dividing the individual permeability values for two gasses A and B:

*α*_A/B_ = *P*_A_/*P*_B_ (3)

The tests were carried out at different temperatures in the range 25–55 °C and at a feed pressure of 1 bar. Gas permeation obeys to an Arrhenius’ law as temperature changes according to the expression:

*P* = *P*^0^ exp(−*E*_P_/*RT*) (4)

where *P*^0^ is a pre-exponential factor, *E*_P_ the apparent activation energy for the permeation.

### 2.5. Scanning Electron Microscopy (SEM)

Samples for morphological analysis were prepared by sputter-coating with a thin film of gold using a QUORUM Q150R S instrument (Quorum Technologies Ltd., Lewes, UK). To analyze the membrane cross-section, some samples were fractured in liquid nitrogen prior to the sputtering. The micrographs were acquired on a Zeiss EVO MA100 Scanning Electron Microscope (Zeiss, Roma, Italy).

## 3. Results and Discussion

### 3.1. CNTs Dispersion in the Polymer Casting Solution

**Figure 3:**
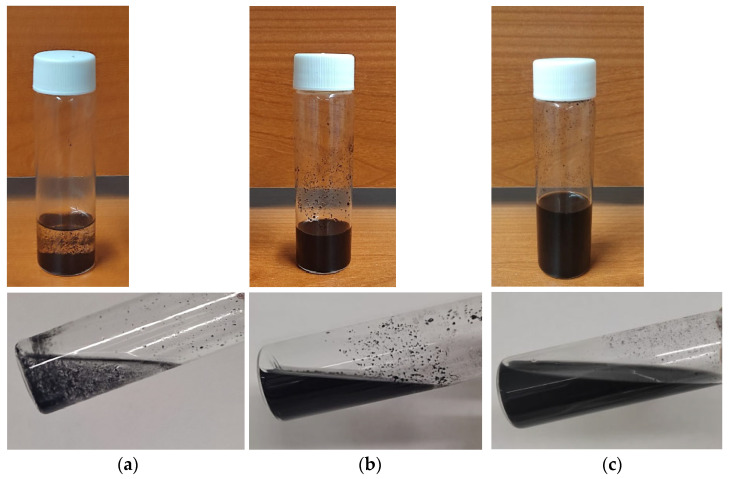
Images of the filler dispersion (casting solution at 12 wt% CNTs). (**a**) Dispersion in ethanol before US; (**b**) dispersion in ethanol after US; (**c**) dispersion in ethanol with the addition of the polymeric solution.

The simple dispersion of the filler in the solvent produces an inhomogeneous suspension in which the CNTs are confined and distinct from the solvent. A prolonged sonication guarantees a certain homogenization of the nanocarbons even after an adequate resting time, without apparent sedimentation of the filler. The addition of the polymer solution to the sonicated slurry of CNTs in ethanol resulted in a good dispersibility of the fillers (Figure 3). Poor tendency to settle by the filler was detected in the latter case. A better behavior was observed when the surfactant was added to the CNTs suspension, even at the highest investigated filler concentration. Indeed, polysorbate surfactants that have long tail groups and unsaturated C-C bonds demonstrated a significant contribution to the stabilization of CNTs dispersions [37].

More concentrated systems probably would have resulted in a worse dispersion of the fillers in the polymer solution even after ultrasonic treatment; in this case, a centrifugation step would have been necessary to achieve satisfactory dispersion of the casting solution.

### 3.2. Morphological Analysis

**Figure 4:**
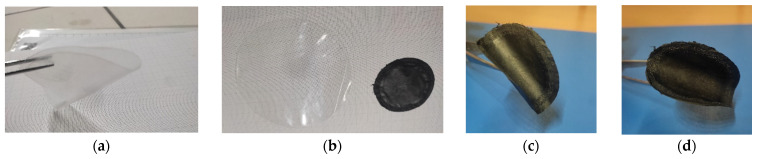
Images of some representative membrane samples: (**a**) membrane based on neat Pebax; (**b**) membrane based on neat Pebax vs. C-9 MMM (Pebax^®^_CNTs 9%); (**c**) C-9 MMM, smooth side; (**d**) C-9 MMM, rough side.

The neat, prepared membranes were transparent and flexible, while the MMMs were black flexible films, opaque and smooth on the side that was in contact with the glass plate during the solvent evaporation and with a quite rough surface on the side directly exposed to air (Figure 4). Differently from the neat Pebax films, which tend to adhere to the glass support used for casting the dope solution, the MMMs containing the CNTs were easily detached from the glass plate. The thickness of the membranes was in the range 70–80 micron with a standard deviation on each sample lower than 5%.

**Figure 5:**
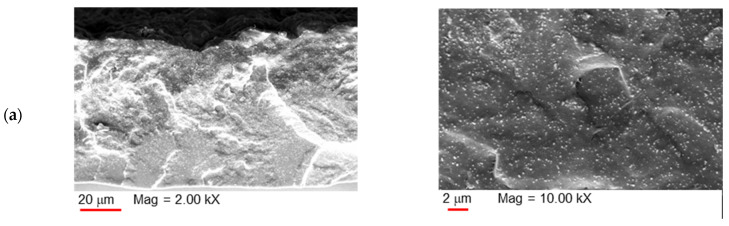

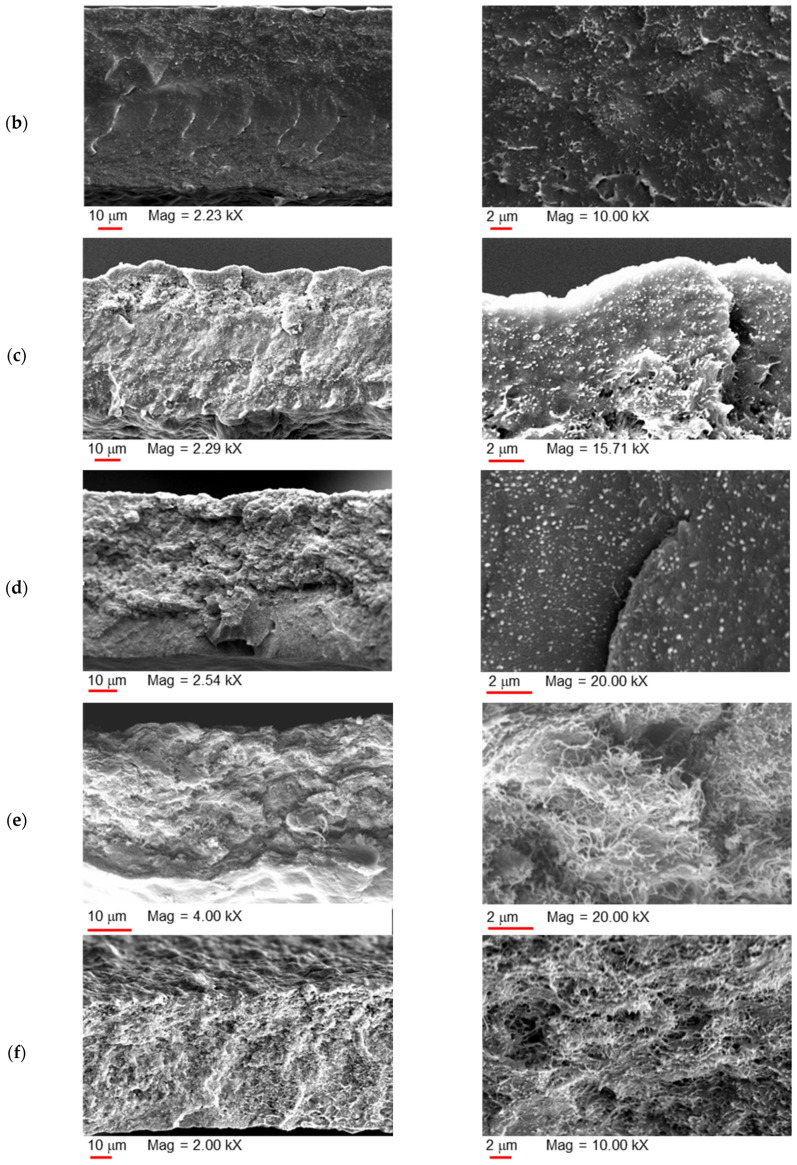
SEM images of the membranes: **left column**: entire cross-section; **right column**: enlarged cross-section. C-3 (**a**); C-6 (**b**); C-9 (**c**); C-12 (**d**); C-18 (**e**); C-24 (**f**). Electron high tension (EHT) = 10 kV; probe current (I probe) = 50 pA; vacuum mode = high vacuum.

The morphological analysis carried out via SEM (Figure 5) highlighted an acceptable dispersion level of nanotubes in the polymer matrix. The samples with a low CNT amount (up to 9 wt%) display an asymmetric structure in which the filler microdomains resulted more concentrated within the top layer, suggesting the migration of CNT microdomains during the solvent evaporation [38]. The concentration of the microdomains was gradually depleted moving from the surface to the bulk of the membrane. Instead, in the more concentrated MMMs, the CNT presence along the membrane cross-section was more uniform. At a high magnification, the CNT bundles become more evident, as well as the presence of some voids in the C-18 and even more in the C-24 samples. Moreover, because of the size of the filler, it was possible to observe only the envelope of CNT bundles and not the individual CNTs.

**Figure 6:**
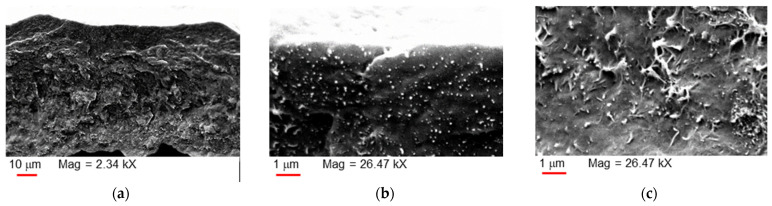
SEM images of the membrane C-12/T80-60: (**a**) cross-section; (**b**) enlarged cross-section (top layer); and (**c**) enlarged cross-section (bulk). Electron high tension (EHT) = 10 kV; probe current (I probe) = 41 pA; vacuum mode = high vacuum.

Figure 6 shows the morphology of the ternary MMM C-12/T80-60 that is similar to that of the samples containing low amount of CNTs. A closer look demonstrated CNT bundles enveloped by the polymer matrix.

### 3.3. FT-IR

**Figure 7:**
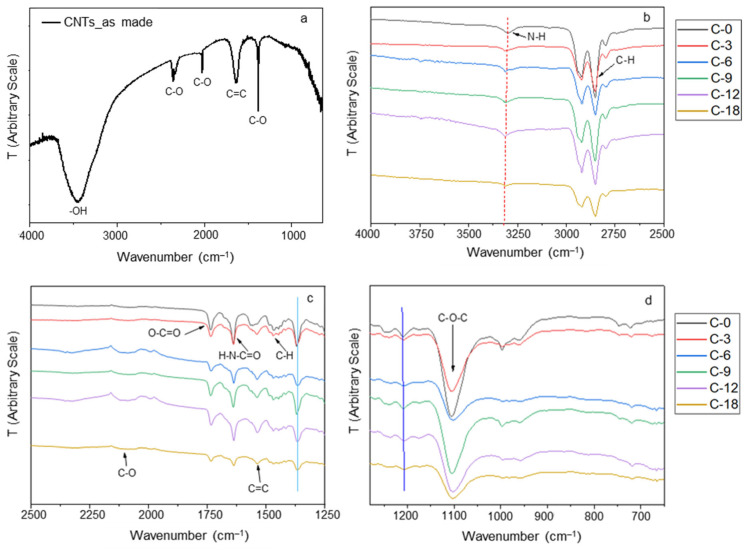
FT-IR-ATR spectra: (**a**) neat MWCNTs; (**b**–**d**) neat Pebax^®^ and MMM films in different wavenumber ranges.

The FT-IR spectra acquired on MWCNTs and on the prepared membranes (pure Pebax^®^ and CNT-loaded samples) are gathered in Figure 7. The spectrum of the MWCNTs (Figure 7a) presented a broad band at 3450 cm^−1^ due to the stretching of the OH groups. Thus, in the not-purified fillers, some -OH groups are present probably because of the presence of the hydrophilic supported catalyst and/or defects on the MWCNTs. The peak at 1636 cm^−1^ corresponds to the stretching mode of the C=C of aromatic rings that shape the CNT framework [39]. The narrow band at ca. 1396 cm^−1^ is due to the presence of C-O bonds.

The spectra recorded on the membranes showed bands at ca. 2853 cm^−1^ (Figure 7b) and 1734 cm^−1^ (Figure 7c) that derive from Pebax and correspond to stretching vibrations of -CH_3_ and O-C=O groups, respectively [40]. The strong band at 1106 cm^−1^ in the neat Pebax^®^2533 film is related to a stretching vibration of the C-O-C (ether group) within the PTMO block (Figure 7c). Those at ca. 1640 and 3300 cm^−1^ are typically assigned to the stretching vibrations for the H-N-C=O and the -NH groups in the polyamide block, respectively. All characteristic polymer bands were maintained in the nanocomposite films, with slight shifts in some MMM samples. A redshift was appreciated in the band at ca. 3300 cm^−1^ (Figure 7b), indicating an interaction of the MWCNTs with the -NH groups in the polyamide block. Interactions between MWCNTs with the polyamide block in Pebax^®^1657 MMMs were also reported [16]. A little redshift was observed for the band at 1464 cm^−1^ that is related the stretching vibration peak of the CH aliphatic group [41] (Figure 7b). The additional peak at 1532 cm^−1^ in the nanocomposite spectra is due to the stretching vibration of C=C in the CNT structure [42] (Figure 7c). Indeed, peaks in the 1450–1650 cm^−1^ spectral region are typically assigned to the stretching vibrations of the C=C group [43]. The small band at ca. 1243 cm^−1^ (C-O-C anti symmetric stretching vibrations of ester group) is related to a possible presence of defects in the nanomaterial structure [44] and has a subtle blue shift.

**Figure 8:**
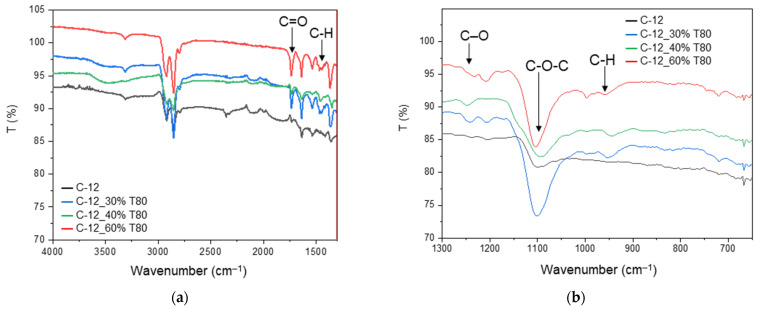
FT-IR-ATR spectra of Pebax^®^-based MMMs containing T80. Transmittance versus wavenumber: (**a**) range 4000–650 cm^−1^, (**b**) enlarged portion, range 1300–650 cm^−1^.

The IR spectra evidenced some differences between the ternary MMMs containing T80 (Figure 8) and the binary CNT-12 MMM. The broad band at ca. 3500 cm^−1^ is due to the stretching vibrations of O-H in polysorbates, while the band at ca. 1740 cm^−1^ is ascribed to the asymmetric C=O stretching vibrations in the used surfactants [45]. The band at 1450 cm^−1^ is related to the C-H bending in CH_2_; the band at 1245 cm^−1^ is due to the stretching of C-O [45]. A more intense band at ca. 1100 cm^−1^ reflects the presence of the C-O-C ether groups in the polymer as well as in the polysorbates. Peaks at ca. 950 and 840 cm^−1^, not present in the binary sample, are also characteristic for C-H deformation in polysorbates [46]. Similar spectra were recorded in the ternary samples containing T20.

These data (from polymer and additives) confirmed interactions among the components of both binary and ternary nanocomposites

### 3.4. TGA

**Figure 9:**
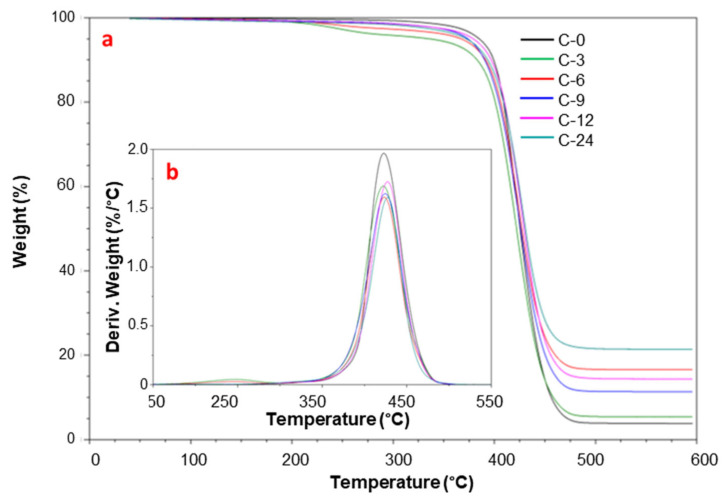
Residual mass (**a**) and mass loss derivative (**b**) as a function of temperature from dynamic TGA tests on the Pebax^®^ and Pebax^®^-CNTs MMMs.

Thermogravimetric analyses (TGAs) were carried out as ramped experiments increasing the temperature range from 40 °C to 600 °C (10 °C/min heating rate) to obtain reliable results on thermal stability of neat Pebax^®^ and its MMMs, comparable with those of literature data [15]. The thermograms and DTG curves of neat Pebax^®^ and its MMMs are shown in Figure 9. The decomposition temperatures reported in Table 2 correspond to the mass losses (TG) at 5% and 50%, to the maximum temperature of thermal degradation (*T*d). The weight residue (%) at 600 °C is reported as well. In order to reduce the uncertainty from the manual determination of the *T*_onset_ tangent point, we considered the temperature at 5% weight loss as *T*_onset_ [40].

The neat Pebax^®^ displayed a single-step degradation, due to the random chain scission mechanism of the main polymer chain [47] that corresponds to 50% weight loss. A decrease in *T*_onset_ was observed in the loaded films, particularly at a relatively low CNTs concentration (3.6 wt%).

The presence of larger amounts of CNTs only slightly affected the thermal degradation pattern of the polymer. No significant differences between neat Pebax^®^ and polymer nanocomposites were observed at temperatures below 350 °C, confirming the thermal stability of the polymer matrix and of the prepared MMMs. The CNTs loading did not influence the degradation temperature, *T*_d_, that displayed values very similar to that of the neat polymer in all MMMs. Specifically, the maximum degradation temperature was at ca. 423 °C for the neat Pebax^®^ and at 422–428 °C for the MMMs, respectively.

The residue of the nanocomposites at 600 °C was larger than that of the neat polymer, confirming the stability of the fillers at this temperature under a nitrogen atmosphere [48]. Preliminary analyses carried out from 40 °C to 800 °C evidenced residue values similar to those observed at 600 °C, with only slight difference of 0.5–2%.

**Figure 10:**
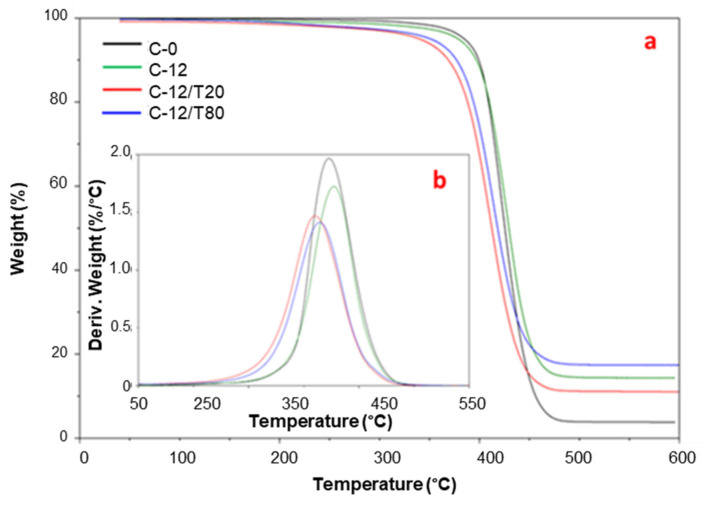
Residual mass (**a**) and mass loss derivative (**b**) as a function of temperature from dynamic TGA tests on the Pebax^®^ and Pebax^®^–12% CNTs membranes containing T20 and T80.

The loading of the additives T20 and T80 to improve the miscibility and stability of the MWCNTs into the polymer matrix induced, compared to C-12, a greater mass loss at both *T*_onset_ and *T*_d_ (Figure 10, Table 3). This is particularly evident with the T20 polysorbate that determined a *T*_onset_ decrease of 45 °C and 39 °C with respect to neat Pebax^®^ and C-12 samples, respectively. A similar trend, but with a minor shift, was registered in the nanocomposites containing the T80 polysorbate.

### 3.5. DSC

**Figure 11:**
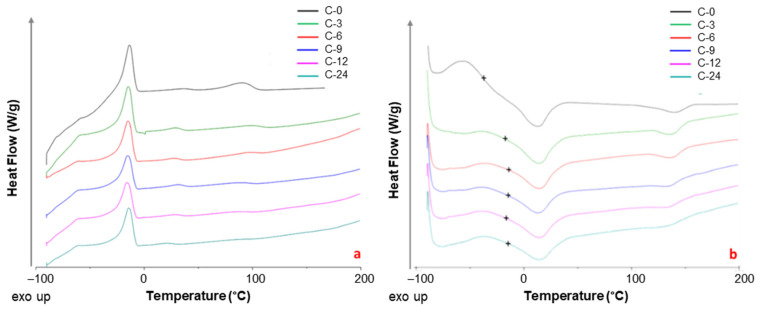
DSC traces of neat Pebax and Pebax/MWCNTs membranes: (**a**) cooling; (**b**) second heating curves are shifted for the sake of clarity. The symbol + represents the *T*_g_ midpoint.

Figure 11 shows the DSC traces obtained during the cooling and second heating on the prepared films. The glass transition temperature (*T*_g_) was determined from the second heating run. Data of melting temperature (*T*_m_) and enthalpy (**Δ***H*_m_) together with crystallization temperature (*T*_c_) and enthalpy (Δ*H*_c_) of both PTMO and PA blocks in neat Pebax^®^ and Pebax^®^-CNT samples are reported in Table 4.

CNTs loading into Pebax^®^2533 matrix did not influence the position of the crystallization peaks in polyether domain, whereas this addition caused a slight crystallization shift to higher temperatures in the PA domain at their low concentrations and a decrease at larger CNTs loadings. The changes observed for the PA domain during the cooling run suggested a preferential interaction of this block with the nanocarbons, as also indicated by the FT-IR analysis. Similar findings were reported in a study on Pebax^®^2533/graphene oxide membranes [49].

The melting peaks related to PTMO and PA12 blocks in the polymer matrix, due to the microphase separation in the block copolymer, are evident for all samples. Comparing the MMMs to neat Pebax^®^, during the second heating run, the main *T*_m_ peak (13.5 °C) for PTMO domain showed a slight increase, while the *T*_m_ peak of PA12 shifted to lower temperatures (from 141 to 137 °C).

A broad glass transition (*T*_g_) at ca. −38 °C was detected for the neat copolymer, with an important increase upon the CNTs addition. The single *T*_g_ that suggests a full homogeneity at the 20–40 nm scale [50] in polymer blends, indicated a good CNT dispersion [51] in the nanocomposites. The positive shift, known as the *T*_g_ enhancement, is a well-documented behavior found in nanocomposites due to the polymer/CNT adhesion [52] that suggests an increasing rigidity of the matrix. Thus, the fillers acted as physical cross-links, restricting the sliding of the polymer chains in the MMMs, similarly to the effect of crystalline regions in semi-crystalline polymers [53].

**Figure 12:**
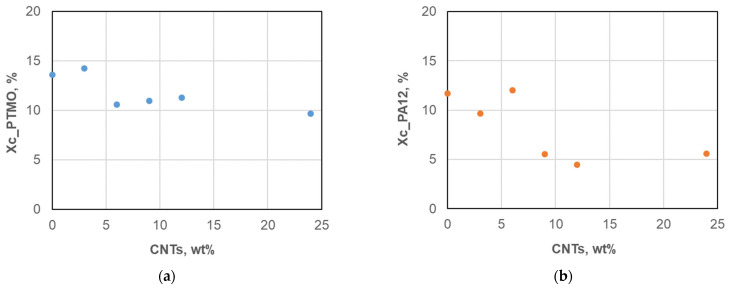
Crystallinity of the Pebax domains evaluated from DSC on the membranes containing different amounts of CNTs: (**a**) PTMO domain; (**b**) PA12 domain.

The FT-IR analysis indicated that the nanocarbons interacted mainly with the -NH group in the amide block of the Pebax matrix. Accordingly, the crystallinity degree of both Pebax domains was reduced in the MMMs samples, particularly for the hard polyamide block (Figure 12). As also reported in other studies [54], despite the large surface area of CNTs that increases crystallite nucleation, the simultaneously restricted mobility of the polymer chains causes a larger resistance to the crystallization process.

The CNTs dispersion raised *T*c in the PA block up to a loading of 6 wt%, indicating a higher nucleation ability of the matrix, as also reported in PEG-CNT nanocomposites [37]. However, at larger-filler loadings, the CNTs bundles could have restricted the folding of some polymeric chains during the crystallization process, resulting in a more pronounced *X*c reduction. The aggregates formed at high nanocarbon loadings enhanced this effect. Instead, a larger crystallinity was found for MMMs containing graphene compared to neat Peba films since graphene nanoplatelets act as nucleating agents being fully exfoliated and well-dispersed [55].

**Figure 13:**
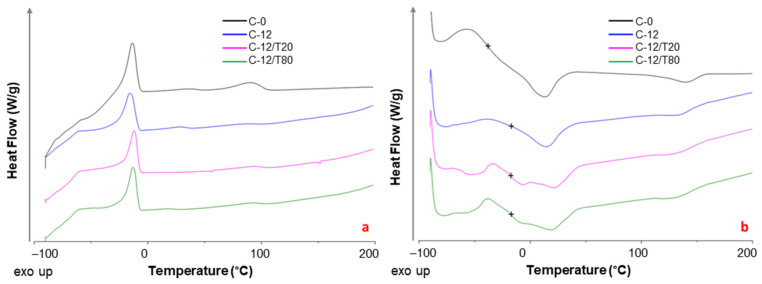
DSC traces of neat Pebax (C-0) and C-12 MMM membranes: (**a**) cooling; (**b**) second heating curves are shifted for the sake of clarity. (**b**) The symbol + represents the *T*_g_ midpoint.

The result of the thermal analysis carried out on the ternary membranes comprising the surfactant-dispersed nanocarbons are shown in Figure 13 and in Table 5.

The ternary MMMs showed a lower *X*c for the PA block than the pristine film (Table 6), in agreement with the previously reported plasticizer effect exerted by the polysorbate in MWNT/PEG nanocomposites [34]. Considering the PA12 block, their behavior was intermediate between the neat copolymer and the binary C-12 sample.

### 3.6. Gas Permeation

#### Binary MMMs

**Figure 14:**
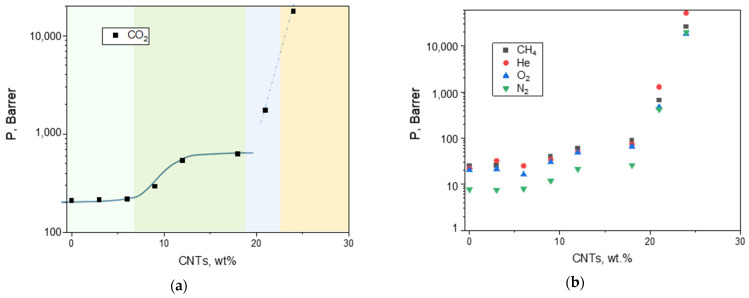
Evolution of the gas permeability measured at 25 °C as a function of the nanocarbons amount (range 0–24 wt%) in Pebax^®^-based films (1 Barrer = 10^−10^ cm^3^ (STP) cm cm^−2^ cmHg^−1^ s^−1^). (**a**) CO_2_; (**b**) CH_4_, He, O_2_, and N_2_. The lines are a guide for the eye. (**a**) The background colors are used to distinguish CNT loadings that result in MMMs having different transport modes.

The gas permeation tests evidenced an enhanced permeability for all gasses upon an increase in the MWCNTs loading in the polymer matrix (Figure 14).

Different molecular gas transport regimes in the membranes can be appreciated in the considered range of nanocarbon loading. Up to a MWCNT concentration of 6 wt%, the MMMs showed gas permeability similar to that measured on the neat Pebax sample. A sharp enhancement was recorded increasing the nanocarbon loading from 9 wt% to 12 wt% that was partially softened in the C-18 membrane. CO_2_ was the more permeable species in the C-0–C-18 samples, while helium became more permeable than the other tested gasses in the C-21 and in C-24 samples.

The percentage gain in permeability reached ca. 300% in the C-18 membrane for CO_2_. The permeability was dramatically enhanced at larger filler loadings, passing from 1700 Barrer (CNT-21) to ca. 18,000 Barrer (CNT-24) in the case of CO_2_. Similar increments were observed also for the other gas species.

In the loading range 0–18 wt%, the shape of the permeability curves (continuous line in Figure 14a) recalls the typical behavior of the electrical conductivity in CNT/polymer nanocomposites that is due to tunnelling through a network of conductive CNT agglomerates [56]. However, the plateau that characterizes the electrical conductivity in CNT based nanocomposites [57] was not kept in the gas permeability plot upon a further increase in the nanocarbon loading (up to 24 wt%).

**Figure 15:**
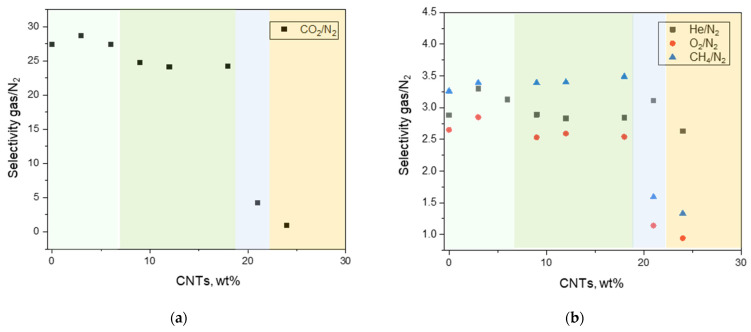
Evolution of the gas/N_2_ permselectivity measured at 25 °C on Pebax^®^-based films containing nanocarbons in the range 0–24 wt%: (**a**) CO_2_/N_2_; (**b**) He/N_2_, O_2_/N_2_ and CH_4_/N_2_. The background colors are used to distinguish CNT loadings that result in MMMs having different transport modes in accordance with Figure 14.

This different behavior suggests a modification in the membrane morphology with increasing CNTs amounts, as supported by the evaluated permselectivity (see SEM images in Figure 5). Interestingly, up to a CNT loading ≤18 wt%, the selectivity was not reduced, even in the MMMs produced at large filler loadings, evidencing a very permeable and virtually defect-free material due to the creation of microvoids (Figure 15). Larger filler amounts disrupted the arrangement of polymer segments, leading to very open structures with a progressively deteriorated permselectivity (e.g., C-21 and CNT-24) in accordance with other studies focusing on Pebax-based nanocomposites [13]. C-24 displayed reduced values, close to that corresponding to Knudsen diffusion mechanism (*α*ij = *P*i/*P*j ≈ (*M*j/*M*i)^0.5^), probably due to filler aggregates within the matrix that cause channeling phenomena capable to enhance the gas flux without discriminating among gas species. Thus, the Knudsen mechanism occurred well above the threshold limit, differently from what reported for MMMs containing CNTs dispersed in a glassy polymer (Knudsen mechanisms just above the percolation limit) [58]. A concomitant solution–diffusion and Knudsen diffusion mechanisms can be inferred from the measured intermediate separation factors in the C-21 membrane. These latter membrane samples are not suited for the separation of gaseous mixtures. However, their gas permeance (ca. 110 GPU for CO_2_ and for O_2_ in C-24) is in the range of that reported for membranes developed for oxygenator applications [59]. Moreover, the used Pebax copolymer is a biocompatible material [40], confirming the possibility of application in this field.

Compared to spherical nanoparticles, the high aspect ratio CNTs typically result in nanocomposite materials having a threshold at low concentration, which corresponds to the creation of a network of CNTs leading to continuous pathways in the polymer matrix [60].

The volume fraction of the MWCNTs in the membrane at the percolation threshold (*C*c, critical concentration) was estimated according to the following expression developed for non-spherical and non-interacting particles [61]:

*C*c ~ *D*/*L* (5)

where *D* is the diameter of the particles [nm], and *L* is the length [nm].

A critical concentration of ca. 0.1% in volume, corresponding to a CNTs loading of 0.09 wt% (CNT density = 0.85 g/cm^3^), was calculated (Equation (5)) for the present case considering bundles having *D* = 20 nm and *L* = 20 micron. However, the CNT concentration level at which gas permeability undergoes a sharp increase is close to 7 wt%, which is higher than that expected for non-interacting fillers, suggesting an influence of CNT waviness and the formation of aggregates in the MMMs as revealed by the SEM analysis. Indeed, CNT waviness has a significant effect on the electrical behavior of nanocomposites. The electrical percolation threshold was found to evidently increase with increasing the waviness degree of CNTs [62].

### 3.7. Diffusion/Solubility

**Figure 16:**
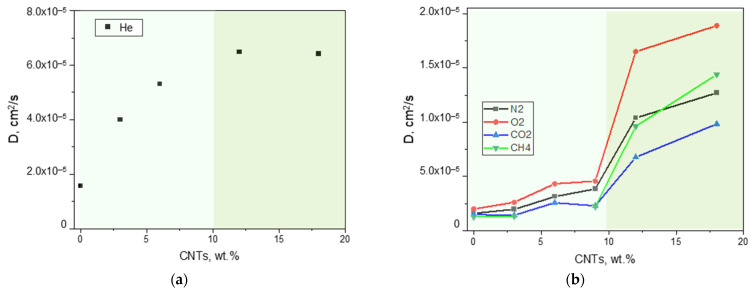
Evolution of the gas diffusion coefficient measured at 25 °C as a function of the nanocarbons amount (range 0–18 wt%) in Pebax^®^-based films. (**a**) He; (**b**) N_2_, O_2_, CO_2_, and CH_4_. The background colors are used to distinguish CNT loadings that result in MMMs having different transport modes.

An in-depth analysis of the permeability contributions revealed that the increased permeability can be attributed to an enhanced apparent gas diffusivity (Figure 16). Thus, the elongated carbon nanofillers insert into polymer chains, creating more free volume available for the gas permeation. Being not purified, we can exclude a gas transport within the CNTs hollow structures.

Another aspect that supports the enhanced permeation flux and gas diffusion is the observed reduced crystallinity evidenced by the DSC analysis. Since, in the temperature range explored for the permeation tests, the PTMO block is in the molten state, the polymer crystallinity resulted only from the PA12 block that was found to reduce more significantly. Therefore, the reduction in crystallinity of the polymer phase was more important than the observed increase in *T*_g_.

The diffusion order of the neat copolymer at a temperature of 25 °C (i.e., He > O_2_ > N_2_ > CO_2_ > CH_4_) that reflects the molecular size of the permeating gasses was kept in the MMMs containing up to 9 wt% CNTs. Instead, C-12 and C-18 MMMs displayed an enhanced diffusion for methane. This unusual behavior (*D*_CH_4_ > *D*_CO_2_) can be related to the lower crystallinity in the MMMs that resulted in a polymeric matrix capable to accommodate the transport of larger molecules.

The gas solubility measured at 25 °C and expressed in [cm^3^_STP_/(cm^3^ cmHg)] was 1.4 × 10^−2^ for CO_2_, 2.0 × 10^−3^ for CH_4_, and 4.8 × 10^−4^ for N_2_ in the neat polymer. In the sample C-12, the solubility was 6.3 × 10^−3^ for CO_2_, 6.4 × 10^−4^ for CH_4_ and 1.6 × 10^−4^ for N_2_. The lower gas solubility observed in the MMMs could be the result of interactions among the membrane components (polymer/nanocarbons), inhibiting potential interaction with gasses, as evidenced for Pebax/Ionic Liquid membranes [63]. However, the high CO_2_/membrane affinity of Pebax is kept also in the MMMs and guarantees an order-of-magnitude-higher solubility of CO_2_ compared to methane. Therefore, despite a reduction in solubility, the enhanced diffusivity did not lower the gas permeability.

#### 3.7.1. Ternary MMMs

**Figure 17:**
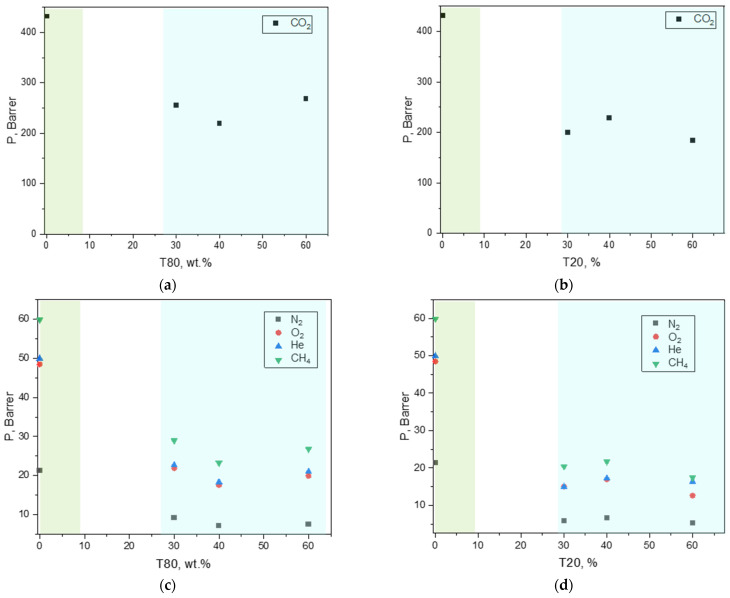
Gas permeability measured at 25 °C on Pebax^®^-based MMMs loaded with CNTs and including T80 or T20. (CNTs/(CNTs + Pebax) = 12 wt%). (**a**,**b**) CO_2_; (**c**,**d**) CH_4_, He, O_2_, and N_2_. The green background refers to the binary MMM (C-12), while the blue background includes the data for the ternary MMMs.

The addition of polysorbates (T20 and T80) to the polymeric matrix produced more permeable samples than the neat polymer films. The same polysorbates were found to enhance gas permeability in Pebax^®^1657 [29,30] and in Pebax^®^2533 [31]. However, the ternary Peba^®^2533/MWCNT MMMs presented a lower permeation flux compared to the binary MMMs containing the same amount of CNTs with respect to the polymeric phase (CNTs/(CNTs + polymer) = 12 wt%) (Figure 17).

**Figure 18:**
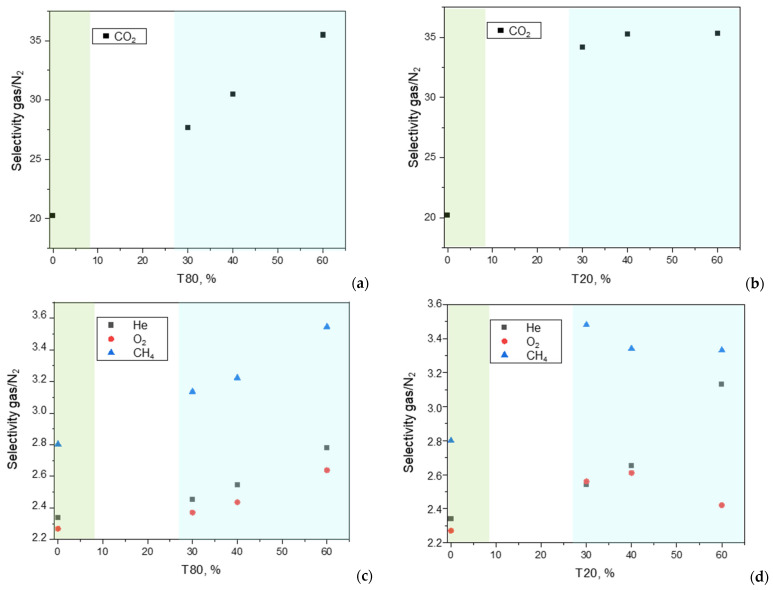
Gas/N_2_ permselectivity measured at 25 °C on Pebax^®^-based MMMs loaded with CNTs and including T80 or T20. (CNTs/(CNTs + Pebax) = 12 wt%). (**a**,**b**) CO_2_/N_2_; (**c**,**d**) CH_4_/N_2_, He/N_2_, and O_2_/N_2_. The green background refers to the binary MMM (C-12), while the blue background includes the data for the ternary MMMs.

On the other hand, an increase in permselectivity was observed in the ternary MMMs (Figure 18) compared to the binary C-12 membrane. These samples have the same polymer/CNTs ratio, but in the ternary MMMs the CNTs percentage on the whole sample is lower compared to the binary C-12. This justifies the permeability behavior as also observed for binary MMMs at concentrations between 3 wt% and 9 wt% compared to C-12 (Figure 14).

**Figure 19:**
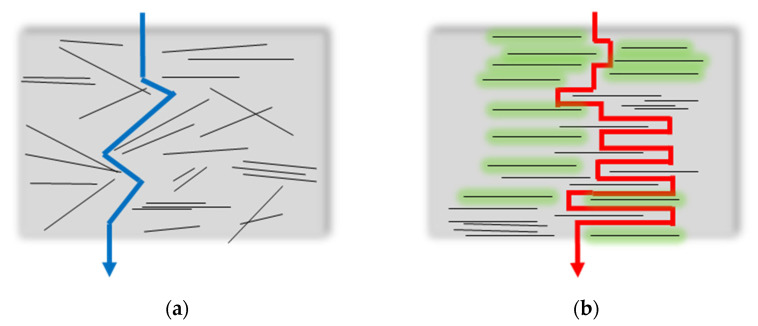
Gas pathways in the MMMs: (**a**) binary MMMs (Pebax/CNT); (**b**) ternary MMMs (Pebax/Polysorbate/CNTs). Lines represent the CNTs; green zones represent the polysorbates in the ternary MMMs.

The permselectivity increment in the ternary MMMs, particularly evident for the CO_2_/N_2_ gas pair, can be explained by the poly (ethylene glycol) chains present in the polysorbate structures that have a high affinity for CO_2_. Moreover, the tendency of CNTs to adsorb surfactant molecules on their outer wall improves their compatibility with the host matrix. This is in agreement with the improved quality of the CNTs suspension once a surfactant was added to them. As a consequence, in the presence of the surfactant, the fillers tend to be better dispersed in the matrix, acting as “obstacles” and creating a more tortuous path to gas permeation (Figure 19).

**Figure 20:**
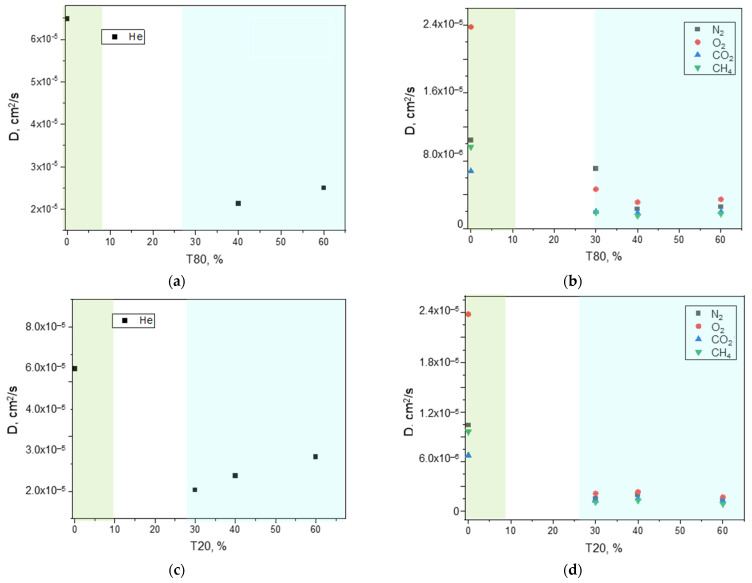
Gas diffusion coefficient measured at 25 °C on Pebax^®^-based MMMs loaded with CNTs (CNTs/(CNTs + Pebax) = 12 wt%) and including T80 or T20. (**a**,**b**) ternary MMMs containing T80; (**c**,**d**) ternary MMMs containing T20. The green background refers to the binary MMM (C-12), while the blue background includes the data for the ternary MMMs.

From a microscopic point of view, the reduced permeability is due to a lower diffusion for all the gasses (Figure 20), as also observed in the binary samples.

Comparing the two polysorbates, that differ in their alkyl chain length, the use of T80 resulted in higher gas permeability than T20 (Figure 17). Indeed, T80 has a longer alkyl chain than T20 (length of 18 or length of 12, respectively), resulting in a more effective increase in the MMM free-volume. In addition, DSC analysis showed that T80 exerted a stronger reduction on the PA crystallinity. Low amounts of T20 were capable to increase the permselectivity that remained almost constant, while in the ternary MMMs containing T80, the gain in selectivity (gas/N_2_) increased with the amount of surfactant.

However, concerning the dispersion of CNTs in the membrane, T80 has a reduced debundling efficiency compared to T20 owing to the bulky hydrophobic groups that can be less suited to penetrate in the nanocarbon aggregates [64].

#### 3.7.2. Temperature Effect on Gas Permeation

Permeation tests were carried out in the temperature range of 25–55 °C on the neat Pebax membrane and on binary and ternary MMMs. The increase in the operative temperature caused an enhanced gas permeability for all gas species, while the selectivity was reduced, as commonly found in polymeric membranes. The data can be represented by the Arrhenius expression (Equation (3)), enabling the determination of *E*_P_ from the slope of the permeability logarithm versus the reciprocal of absolute temperature (Table 7).

The order for the activation energy of the different gasses in the Pebax matrix was preserved in the MMMs: larger activation energy corresponds to larger and slow molecules (e.g., N_2_). Carbon dioxide, instead, being linear and permeable, had the lowest value of activation energy in all the tested samples. Thus, the effect of the temperature was more significant for less permeable gasses, leading to a reduction in the selectivity (e.g., CO_2_/N_2_).

Reduced *E*p values were measured in the more permeable MMMs compared to the neat polymer, suggesting a more open structure, less sensitive to temperature changes.

Compared to binary C-12 membrane, the ternary MMMs showed similar activation energy values at the lowest surfactant content. Furthermore, a lower activation energy was observed in the ternary films containing increasing amounts of the surfactant, thus implying a diminished resistance for permeation of gas molecules, particularly in the case of CO_2_. This is in agreement with a previous study on membranes based on Pebax^®^1657 and the polysorbates T20 and T80 that evidenced a reduced activation energy for diffusion [29].

## 4. Literature Comparison

To the best of our knowledge, only a few studies focused on MMMs based on Pebax^®^2533. The majority of them investigated the more rigid Pebax^®^1657copolymer, due to the better intrinsic selectivity of such a more rigid copolymer. A study on MMMs based on Pebax^®^ 1657 loaded with SWNTs or MWNTs reported a little impact on the selectivity at a filler loading ≤5 wt% and a drop for both CO_2_ permeability and selectivity at filler concentrations above 5 wt% [17].

The Pebax^®^ 2533 copolymer grade used in this work comprises a soft polyether block as the main phase (80%). Therefore, the Pebax^®^2533 matrix, more flexible than the 1657 grade, is capable to incorporate a quite large amount of nanofillers without losing the separation performance. Indeed, MMMs based on MWCNTs dispersed in the glassy polyethersulfone (PES) showed marginal increase in the N_2_ and CO_2_ permeability as the filler loading increased from 2 to 10 wt%, while the selectivity was deteriorated [65]. On the contrary, a plateau was reached in PMMA and PVTMS, indicating an agglomeration of the used MWCNTs [66].

A comparison with the literature data of several Pebax^®^2533-based MMMs is proposed in Table 8, showing that the present binary Pebax/MWCNT samples are characterized by the highest permeability, while interesting selectivity can be achieved using the ternary MMMs.

Remarkable aspects of the developed membranes are related to the use of ethanol as the solvent for the membrane preparation. Indeed, ethanol was selected due to its non-toxicity compared to other alcohols that display similar solvent strength, polarity, and hydrogen-bonding ability, such as methanol and isopropanol [67].

Moreover, the use of not-purified and unfunctionalized fillers significantly reduces the costs of the filler production, suggesting this filler–polymer combination as an appropriate way to enhance the gas transport properties of the starting copolymer material. Surfactant’s effectiveness in improving the dispersion of the filler in solution could be exploited to upscale the preparation of nanocomposites to also be used in different applications.

The use of different Pebax grades to incorporate MWCNTs could improve the separation performance of the MMMs, as recently reported for Pebax^®^1074/MWCNT membranes [68]. The MMMs based on Pebax^®^1074, more rigid than Pebax^®^2533 (45 wt% of PA12), showed an increase in both permeability and selectivity for CO_2_/N_2_ separation. However, we proved the possibility to prepare self-standing MMMs with a high filler concentration (up to 24 wt%), while in the Pebax^®^1074, MWCNT loadings larger than 12 wt% lead to too fragile samples [68].

**Figure 21:**
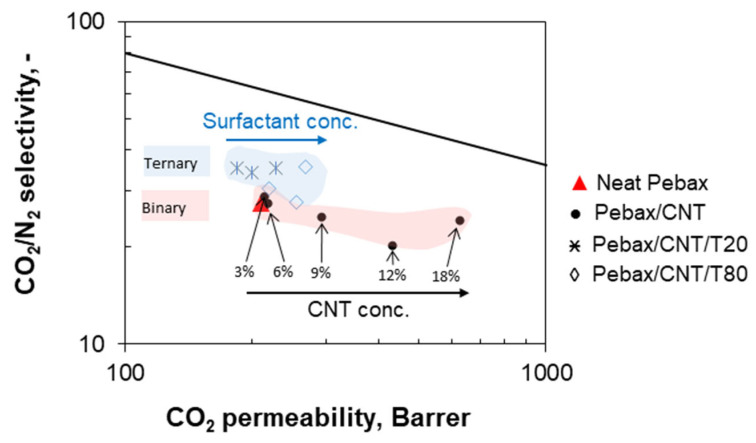
Robeson plot for the CO_2_/N_2_ separation showing the permeation parameters measured at 25 °C on Pebax^®^-based binary MMMs loaded with CNTs (0–18 wt%) and ternary MMMs (CNTs/(CNTs + Pebax) = 12 wt% and including T80 or T20). The upper bound is the same as that reported in 2008 by Robeson [69].

Figure 21 shows the permeation parameters measured at 25 °C on binary and ternary MMMs in a Robeson plot for the CO_2_/N_2_ separation [59]. The performance of the binary MMMs moved towards the upper bound since the CNT addition increased permeability and kept the intrinsic selectivity of the polymer matrix. On the other hand, both an improved permeability and selectivity can be obtained in the ternary MMMs.

**Table 8:** Comparison of the performance of Pebax^®^2533 MMMs for CO_2_/N_2_ separation.

Polymer/Filler	Loading (wt%)	Permeability (Barrer) CO_2_	Ideal Selectivity (-) CO_2_/N_2_	T (°C)	Feed Pressure (bar)	Ref.
2533/ZIF-8	0	62	25	25	6	[70]
	2	84	34			
	4	117	44			
	8	158	51			
	16	184	33			
2533	0	125	18	24	2	[71]
2533/ZIF-8	10	225	33			
2533/ZIF-8/PEI	10	230	35			
2533/ZIF-8/PEI@IL	10	220	43			
2533/Pluronic P123 (surfactant)/ZIF-8	5 ZIF-8 2.5 P123	328	19.5	45	4	[72]
2533	0	365	23.8	35	1	[49]
2533/ Graphene oxide (GO)	0.02	371	24.0			
2533/porous GO (PGO)	0.02	397	23.8			
2533/Polyetheramine functionalized GO (PEAGO)	0.02	380	24.2			
2533	0	210	27.4	25	1	This work
2533/MWCNT Binary	3	214	28.7			
	6	218	27.4			
	9	292	24.7			
	12	536	24.1			
	18	626	24.2			
2533	0	169	22.5	25	0.6	[31]
2533/T20	15	179	25.9			
	35	210	28.4			
	50	223	31.0			
	65	267	36.6			
2533/T80	15	200	32.0			
	35	219	29.4			
	50	240	36.9			
	65	289	40.7			
2533/MWCNT Ternary	C-12/T80-60	268	35.5	25	1	This work
	C-12/T20-60	184	35.3			

## 5. Conclusions

Flexible free-standing films based on Pebax^®^2533 loaded with different amounts of low-cost MWCNTs (range 3–24 wt%) are successfully obtained according to a solution casting procedure employing a non-toxic solvent (ethanol). Mechanical dispersion methods, such as ultrasonication, are investigated as well as the chemical methods (addition of a surfactant) to disperse the filler particles in the casting solution.

FT-IR analysis of pure Pebax^®^ and CNTs-loaded samples suggests preferential interactions of the fillers with the amide block of the polymer matrix. Thermal stability of the copolymer matrix, as measured by dynamic TGA, is preserved in the binary composites and slightly reduced in the ternary samples. The differential calorimetry indicates a good MWCNTs dispersion in the matrix. The observed *T*_g_ enhancement indicates a higher interfacial interaction between the polymer matrix and the loaded fillers in the MMMs, inhibiting the crystallization of the copolymer blocks. These findings are consistent with the steadily improved gas permeability upon the addition of MWCNTs (up to 17,600 Barrer for CO_2_ in the sample at 24 wt%). A nonlinear change in permeability is obtained with a sharp increase between 6 and 12 wt% of MWNT loading. The percentage variation in permeability in the loaded films with respect to the neat polymer (*P*/*P*_0_) reaches a value of 300% at a CNTs concentration of 18 wt% in the membrane.

The gas permeation data unveil the membrane microscopic structure. The nanocarbon loading could generate microvoids within the polymer matrix that make it more permeable but still selective to gasses up to a CNTs concentration of 18 wt%. The permselectivity is reduced at higher filler concentration. The lower apparent activation energy for permeability indicates the presence of additional pathways in the MMMs. The observed enhanced permeability is due to interfacial regions that form open channels through the polymer film with substantially increased gas diffusivity. Indeed, the gas diffusion increases in the MMMs with CNTs owing to the insertion of the elongated fillers disturbing the polymer chain packing. Peculiar behaviors can be appreciated at loadings ≥12 wt% (enlarged gas diffusion for bulkier molecules as methane). The ternary MMMs containing the nanocarbons and a polysorbate are even more permselective than the binary MWCNT–polymer membranes, confirming the beneficial effect of the surfactant. Therefore, the use of such surfactants can be suggested for large-scale production of CNT-based MMMs to improve the filler dispersion.

## Figures and Tables

**Table 1:** Codes and composition of the ternary MMM samples.

Membrane Code	[CNT/(Peba + Polysorbate + CNT)] (wt%)	[Polysorbate/(Peba + Polysorbate + CNT)] (wt%)
C-12/T80-30	8	30
C-12/T80-40	6	40
C-12/T80-60	4.5	60
C-12/T20-30	8	30
C-12/T20-40	6	40
C-12/T20-60	4.5	60

All the ternary membranes were prepared using a ratio CNTs/(Peba + CNTs) = 12 wt%.

**Table 2:** Thermogravimetric data of neat Pebax^®^ and its MMMs.

Sample	T_Δm = 5%_ (°C) ^a^	T_Δm = 50%_ (°C) ^b^	T_d_ (°C) ^c^	% R ^d^ (600 °C)
C-0	385.1	425.5	422.7	3.8
C-3	335.2	422.0	422.4	5.4
C-6	367.2	427.2	423.6	16.6
C-9	373.8	426.0	424.8	11.3
C-12	379.2	429.4	427.6	14.4
C-24	372.4	430.7	427.6	21.4

a: Onset of degradation (temperature of 5% weight loss); b: Onset of degradation (temperature of 50% weight loss); c: Decomposition maximum temperature of thermal degradation; d: Weight residue (%) at 600 °C.

**Table 3:** Thermogravimetric data of neat Pebax^®^ and its ternary MMMs.

Sample	T_Δm= 5%_ (°C) ^a^	T_Δm = 50%_ (°C) ^b^	T_d_ (°C) ^c^	% R ^d^ (600 °C)
C-0	385.1	425.5	422.7	3.8
C-12	379.2	429.4	427.6	14.4
C-12/T80	351.4	418.2	414.8	17.4
C-12/T20	340.4	412.3	410.3	11.0

a: Onset of degradation (temperature of 5% weight loss); b: Onset of degradation (temperature of 50% weight loss); c: Decomposition maximum temperature of thermal degradation; d: Weight residue (%) at 600 °C.

**Table 4:** Glass transition temperature (*T*_g_), melting (*T*_m_) and crystallization (*T*_c_) temperatures, enthalpy of fusion (Δ*H*_m_) and enthalpy of crystallization (Δ*H*_c_) of PTMO and PA blocks in the cooling and second heating scans of neat Pebax^®^ and its nanocomposite membranes.

Membrane Sample	Cooling				II Heating				
	Polyether Domain		Polyamide Domain		Polyether Domain			Polyamide Domain	
	*T*_c_ (°C)	Δ*H*_c_ (J/g)	*T*_c_ (°C)	Δ*H*_c_ (J/g)	*T*_g_ (°C)	*T*_m_ (°C)	Δ*H*_m_ (J/g)	*T*_m_ (°C)	Δ*H*_m_ (J/g)
C-0	−14.1	35.74	90.8	8.45	−37.7	13.5	21.40	140.9	5.78
C-3	−14.8	31.55	94.8	2.49	−16.5	14.8	21.75	139.5	4.61
C-6	−14.8	24.18	94.1	1.34	−14.5	15.5	15.68	139.8	5.56
C-9	−15.1	21.51	85.7	1.29	−14.9	14.6	15.70	138.5	2.48
C-12	−15.8	22.83	78.3	0.80	−16.3	15.4	15.60	137.1	1.94
C-24	−14.1	23.11	-	-	−14.9	14.9	13.34	138.8	2.08

**Table 5:** Glass transition temperature (*T*_g_), melting (*T*_m_) and crystallization (*T*_c_) temperatures, enthalpy of fusion (Δ*H*_m_), and enthalpy of crystallization (Δ*H*_c_) of PTMO and PA blocks in the cooling and second heating scans of neat Pebax^®^ and Pebax/MWCNTs_12% membranes.

Sample	Cooling				II Heating				
	Polyether Domain		Polyamide Domain		Polyether Domain			Polyamide Domain	
	*T*_c_ (°C)	Δ*H*_c_ (J/g)	*T*_c_ (°C)	Δ*H*_c_ (J/g)	*T*_g_ (°C)	*T*_m_ (°C)	Δ*H*_m_ (J/g)	*T*_m_ (°C)	Δ*H*_m_ (J/g)
Pebax^®^2533	−14.1	35.74	90.8	8.45	−37.7	13.5	21.40	140.9	5.78
Pebax^®^-CNT_12 wt%	−15.8	22.83	78.3	0.80	−16.3	15.4	15.60	137.1	1.94
Pebax^®^-CNT_12 wt%_T20	−11.7	19.81	90.6	1.72	−16.9	24.7	9.56	135.5	2.58
Pebax^®^-CNT_12 wt%_T80	−12.8	21.36	86.6	1.40	−16.4	22.0	12.18	132.8	1.28

**Table 6:** Crystallinity degree (*X*c, %) calculated for the PTMO and PA blocks in the neat sample and in binary and ternary MMMs containing MWCNTs ((filler/polymer + filler) = 12 wt%).

	Crystallinity Degree (%)			
Sample	Cooling		II Heating	
	Polyether Domain	Polyamide Domain	Polyether Domain	Polyamide Domain
Pebax^®^2533	22.7	17.2	13.6	11.7
Pebax^®^-CNT_12 wt%	16.5	1.8	11.3	4.5
Pebax^®^-CNT_12 wt%_T20	23.3	6.5	11.3	9.7
Pebax^®^-CNT_12 wt%_T80	25.1	5.3	14.3	4.8

**Table 7:** Activation energy for permeability (*E*_P_) in the neat Pebax membrane and on the binary and ternary MMM samples.

Membrane Code	Activation Energy for Permeability, *E*_p_ (kJ/mol)				
	He	CO_2_	CH_4_	O_2_	N_2_
C-0	27.3	18.9	32.8	30.2	38.3
C-9	25.0	18.2	29.8	28.3	33.1
C-12	29.6	18.7	32.1	28.2	30.5
C-18	24.4	15.8	26.3	26.6	29.8
C-12/T80-30	29.7	18.5	31.3	29.8	30.9
C-12/T80-40	26.0	17.6	30.0	28.5	31.8
C-12/T80-60	24.9	16.8	29.3	28.9	33.2
C-12/T20-30	30.5	19.9	33.6	32.4	37.9
C-12/T20-40	26.5	16.7	30.2	27.0	31.9
C-12/T20-60	22.8	18.1	29.7	29.4	32.4

## Data Availability

The original contributions presented in this study are included in the article. Further inquiries can be directed to the corresponding author.
